# Identification of Biological Pathways Contributing to Marbling in Skeletal Muscle to Improve Beef Cattle Breeding

**DOI:** 10.3389/fgene.2019.01370

**Published:** 2020-02-07

**Authors:** Zahra Roudbari, Susan L. Coort, Martina Kutmon, Lars Eijssen, Jonathan Melius, Tomasz Sadkowski, Chris T. Evelo

**Affiliations:** ^1^Department of Animal Science, Faculty of Agriculture, University of Jiroft, Jiroft, Iran; ^2^Department of Bioinformatics-BiGCaT, NUTRIM School of Nutrition and Translational Research in Metabolism, Maastricht University, Maastricht, Netherlands; ^3^Maastricht Centre for Systems Biology (MaCSBio), Maastricht University, Maastricht, Netherlands; ^4^Department of Physiological Sciences, Faculty of Veterinary Medicine, Warsaw University of Life Sciences - SGGW, Warsaw, Poland

**Keywords:** marbling, curation pathway for cow, signaling pathway, improve breeding selection, transcriptomics profiling

## Abstract

Red meat is an important dietary source that provides part of the nutritional requirements. Intramuscular fat, known as marbling, is located throughout skeletal muscle. Marbling is a trait of major economic relevance that positively influences sensory quality aspects. The aim of the present study was to identify and better understand biological pathways defining marbling in beef cattle. Pathway analysis was performed in PathVisio with publicly available transcriptomic data from semitendinosus muscle of well-marbled and lean-marbled beef. Moreover, for *Bos taurus* we created a gene identifier mapping database with bridgeDb and a pathway collection in WikiPathways. The regulation of marbling is possibly the result of the interplay between signaling pathways in muscle, fat, and intramuscular connective tissue. Pathway analysis revealed 17 pathways that were significantly different between well-marbled and lean-marbled beef. The MAPK signaling pathway was enriched, and the signaling pathways that play a role in tissue development were also affected. Interestingly, pathways related to immune response and insulin signaling were enriched.

## Introduction

Red meat is as an important dietary source that provides part of the nutritional requirements such as proteins, minerals, B-complex vitamins, and essential fatty acids (McAfee et al., 2010). Control of meat quality is very important for meat producers and meat sellers to satisfy customer’s preferences (Bernard et al., 2007). Marbling, a trait that describes the presence intramuscular fat, is of major economic relevance for beef producing cattle that has a positive impact on sensory quality traits, such as flavor, juiciness, and tenderness of meat. Studies have shown that marbling depends on factors such as breed, genotype, age, diet, husbandry, and growth stages. Although in marbling the environmental factors play an important role, the genetic background of the animals is the major factor defining the marbling status (Yamada et al., 2006). O’Connor et al. studied the effect of breed-type on marbling, their results demonstrated that increase in meat marbling from *Bos taurus* cattle (Hereford, Red Angus, Angus, and Tarentaise breeds) can increase the tenderness, more than the Bos indicus cattle (Braford, Red Brangus, and Simbrah breeds) (O’Connor et al., 1997). Although, Shackelford et al., found that, the lower tenderness of meat from Bos indicus cattle is mainly because of decreased postmortem proteolysis which result from elevated calpastatin activity that the possibility existed for an interaction between breed and the influences of marbling score on tenderness (Shackelford et al., 1991). Also, Wulf et al., reported that, in Charolais and Limousin breeds, marbling correlated with calpastatin activity and shear force. He suggested that selection for increased marbling based on these genetic effects, in those two breeds, might be effective for enhancing beef tenderness (Wulf et al., 1996). There is substantial evidence from transcriptomics studies that gene expression profiles affect phenotypic variation for marbling (Cesar et al., 2015). Understanding the signaling pathways that make up the regulatory network in the marbling process can help steer the breeding process (Thaller et al., 2003). Therefore, animal breeding specialists have attempted system-oriented approaches to investigate major economic traits (Lee et al., 2010). It has been shown that marbling differences may be a function of a number of complex interactions among biological pathways. Therefore, a pathway analysis with differential gene expression patterns can result in a better understanding of muscle physiological states and their influence on beef quality and animal welfare (Hocquette et al., 2012). The public availability of transcriptomics data from beef producing cattle, provides new opportunities to explore the global gene expression in muscle to investigate physiological processes and their influence on meat sensory quality traits (Lee et al., 2010; Hocquette et al., 2012).

Within the genomic region of marbling there are several genes considered as parts of QTLs such as EDGPR1, Titin, Akirin 2, and RPL27 (Takasuga et al., 2007) which were mapped in a half-sib family of Japanese Black cattle (Yamada et al., 2006). Thus, these genes were considered as positional functional candidates for the genes responsible for marbling. This study aims at identifying genes and biological pathways regulating marbling of muscle tissue in beef cattle based on publicly available transcriptomics data obtained from a study by Sadkowski and coworkers (2014). We updated and extended the pathway collection for *B. taurus* at WikiPathways (Slenter et al., 2017) an online pathway repository, and a *B. taurus* gene product identifier mapping BridgeDb database was created to allow mapping of expression data to the gene databases identifiers used in the pathways (van Iersel et al., 2010). Sadkowski et al., 2014 measured global gene expression in skeletal muscle of three cattle breeds, i.e., Limousin, Holstein-Friesian, and Hereford, using Agilent microarray chips. Pathway and network analysis were performed to select the important biological pathways involved in marbling and their interactions.

## Materials and Methods

### Transcriptomics Data Set

The study by Sadkowski et al., 2014 compared gene expression in semitendinosus skeletal muscle of well-marbled beef (Holstein-Friesian and Hereford) versus lean-marbled beef (Limousin). Their publicly available microarray data set was used in the present study (NCBI GEO GSE46411). The Holstein-Friesian, Hereford, and Limousin groups consisted of four animals each. Samples for total RNA isolation were taken instantaneously after slaughter from semitendinosus muscle and were kept in liquid nitrogen for transportation and then at −80°C until analyzed. Quality of RNA samples was evaluated using Bioanalyzer 2100 (Agilent Technologies, USA). Only samples with RIN ≥ 8 were further analyzed (Sadkowski et al., 2014).

### Agilent Microarray Data Analysis

Global gene expression was measured with Agilent Two-Color Mi Bovine (V2) 4 x 44K Gene Expression Microarray oligonucleotide slides (Agilent, USA). Sadkowski and coworkers checked the quality of the data and performed LOWESS normalization. The normalized transcriptomic data compared well-marbled beef Holstein-Friesian (n = 4) or Hereford (n = 4) to lean-marbled beef (n = 4). The four log10 fold change (log10FC) values for each group comparison were averaged to obtain an estimate of the 10logFC between the entire groups. Furthermore, a one-sample t-test was performed on both sets of four values, comparing those to 0 (giving a p-value indicating the significance of these values being different from 0 = no change). Bovine genes were considered to be significantly, differentially expressed with p ≤ 0.05 and an absolute FC ≥ 1.3 (Sadkowski et al., 2014).

### *B. taurus* Pathway Collection

The online biological pathway repository, WikiPathways (Slenter et al., 2017), contains pathways of different species, however a *B. taurus* collection was missing. We updated and extended the pathway collection for *B. taurus*. We also created a *B. taurus* gene identifier (ID) mapping database based on mappings present in the Ensembl-based BridgeDb framework (van Iersel et al., 2010). The newly created *B. taurus* ID mapping database was used to annotate genes and proteins in pathways from WikiPathways and to perform pathways analysis. A online and freely available version of the database for the Ensembl build 85 is accessible at (http://bridgedb.org/data/gene_database/archive/r85/Bt_Derby_Ensembl_85.bridge.zip). Second, the WikiPathways homology based the homology mapper which is available at GitHub (https://github.com/PathVisio/homology.mapper) was updated to improve homology coverage for gene products that were annotated with different data sources. The pathways were converted from human pathways, with a required minimum successful conversion of at least 50% of the original human genes. Third, we manually curated all converted pathways to check whether the genes were correctly annotated and pathways are relevant in *B. taurus*. Finally, new pathways directly derived from cow breeding literature and not present in the WikiPathways collection were designed in PathVisio (v3.2.0) (Kutmon et al., 2015), the pathway creation, visualization, and analysis tool. All pathways were uploaded in gpml format to WikiPathways using the WikiPathways plugin (https://www.pathvisio.org/plugin/wikipathways-plugin/) for PathVisio.

### Pathway-Based Over-representation Analysis

To analyze and visualize the molecular changes in marbling at biological process level a pathway-based over-representation analysis was performed in PathVisio (v3.2.0) (Kutmon et al., 2015). The *B. taurus* WikiPathways pathway collection, containing 286 pathways (6/30/2015), and the *B. taurus* ID mapping database, was used in the analysis. The pathways are ranked based on a standardized difference score (Z-score) based on the expected value and standard deviation of the number of significantly (p ≤ 0.05) and differentially (absolute FC ≥ 1.3) expressed genes in a pathway. Biological pathways significantly changed when (i) Z-score > 1.96, (ii) permuted p-value < 0.05 and (iii) minimum number of changed genes is 3. Additionally, alterations in gene expression (log10FC and p value) when comparing Hereford to Limousin were visualized on the *B. taurus* pathways with PathVisio.

### Gene Ontology Overrepresentation Analysis

To find the biological processes in which differentially expressed genes were over represented while no pathways for these processes were present in the *B. taurus* collection at WikiPathways we performed Gene Ontology (GO) analysis *via* the GO-Elite web-interface (Zambon et al., 2012). GO-Elite is a flexible tool for GO-based over-representation analysis. To identify GO processes the following settings in GO-Elite were used: (i) 2000 permutations, (ii) Z-score GO pruning algorithm, (iii) Z-score threshold >1.96, (iv) p-value threshold <0.05 and (v) minimum number of changed genes is 3 (apart from the method specific permutations those are the same criteria as used for the pathway analysis). This approach not only helps to unify the characteristics and functions of the genes but also to attain a broader perspective of the muscle physiological processes and their influence on meat quality.

### Integrated Network Analysis

To visualize the pathway and GO analysis results and their interactions the network analysis and visualization tool, Cytoscape (version 3.2.0), was used (Shannon et al., 2003). First, all enriched pathways and the differentially expressed genes present in these pathways were selected. Second, all changed GO processes and the Differentially Expressed genes present in these GO classes were selected. Third, both results were combined into one network showing the interaction between pathways and GO classes based on corresponding differentially expressed genes. Finally, differences in gene expression between well-marbled and lean-marbled skeletal muscle were visualized in the network.

## Results

### Identification of Differentially Expressed Genes Between Well-Marbled and Lean-Marbled Skeletal Muscle

In the selected transcriptomic data set of beef marbling 42,990 microarray reporters were measured in both lean marbling beef (Limousin) and well marbling beef (Hereford and Holstein-Friesian) animals. Statistical analysis was performed on 29,677 reported genes that remained from the 42,990 reporters after quality control and annotation with Ensembl gene IDs. In the Hereford breed compared to the Limousin breed, 1,513 were higher expressed and 1,556 lower expressed (absolute log10FC >0.11 and p-value <0.05). When comparing the Holstein-Friesian breed to the Limousin breed, 1,772 genes were higher expressed and 2,458 lower expressed in the Holstein Friesian breed. The genes that met these criteria were used for further analysis.

### Creating of *B. taurus* Pathway Collection and Pathway Design

**Figure 1:**
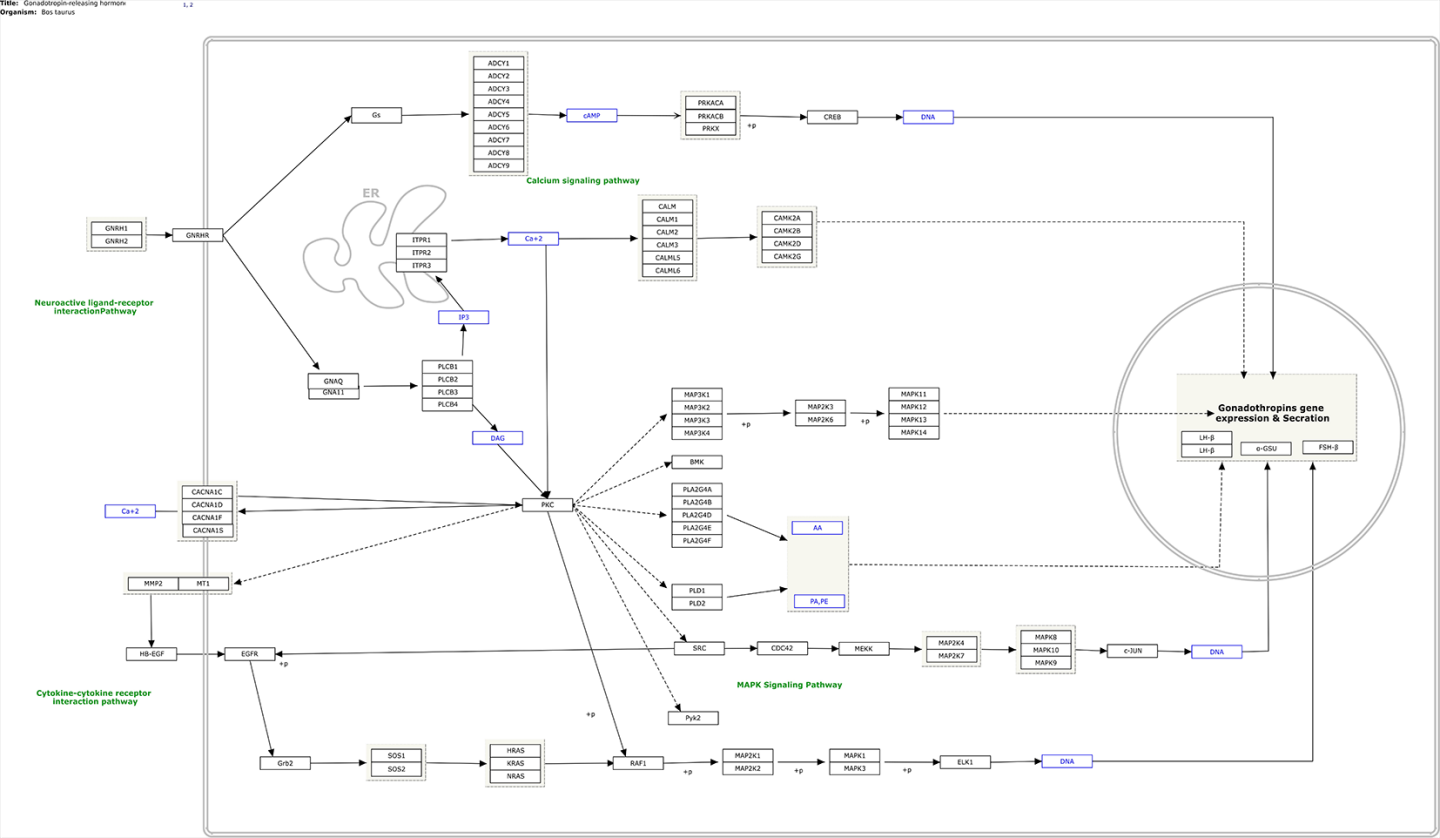
Gonadotropin-releasing hormone (GnRH) signaling (*B. taurus*) (based on Widmann et al., 2013) and available at https://www.wikipathways.org/instance/WP2901.

In total, 282 human pathways were converted from human pathways to cattle pathways. All these pathways were manually checked and are available at (https://www.pathvisio.org/downloads/download-pathways/). Moreover, 4 pathways were newly created based on the bovine breeding literature: Growth hormone signaling (WP2890) (Roudbari Z and Kutmon M: Growth Hormone (GH) Signaling (*B. taurus*); (https://www.wikipathways.org/instance/WP2890), Growth hormone receptor signaling (WP2891) (Roudbari Z, Hanspers K, Evelo C, Kutmon M: Growth Hormone Receptor (GHR) Signaling (*B. taurus*) (https://www.wikipathways.org/instance/WP2891), IGF1-signaling (WP2892) (Roudbari Z, Evelo C, Willighagen E, Mélius J, Hanspers K, Kutmon M: IGF1-signaling (*B. taurus*) (https://www.wikipathways.org/instance/WP2892), and Gonadotropin- releasing hormone signaling (WP2901) (Roudbari Z, Kutmon M, Pico A, Willighagen E, Mélius J: Gonadotropin-releasing hormone (GNRH) signaling (*B. taurus*) (https://www.wikipathways.org/instance/WP2901). As an example, the newly designed GNRH signaling pathway is shown in **Figure 1**. The elements of this process are the key factors stimulating gonadotropin release from the pituitary, which controls the release of luteinizing hormone and follicle-stimulating hormone, and reproductive development in mammals.

### Pathway Analysis

When comparing Hereford with Limousin breed ten biological pathways that were formerly known to be involved in marbling (Cui et al., 2012; Lim et al., 2013; Silva-Vignato et al., 2017) were found to be significantly enriched in differentially expressed genes (z-score > 1.96) (**Table 1**). Four biological processes such as: The Hypertrophy Model, P38 MAPK signaling, IL-1 signaling, and insulin signaling pathways, which are known to be important in marbling development are described in more detail, and the pathways are shown in Results section. Interestingly, some pathways not yet known to play a role in marbling were also found to be enriched in differentially expressed genes when comparing Hereford with Limousin breed. These included histone modifications and vitamin D metabolism pathways, in addition to Hereford breed that is used for meat production, the Holstein-Friesian breed that is a dairy cattle, was also compared with the Limousin breed. Pathway analysis revealed that some but not all of the marbling related pathways found for the comparison between Hereford breed and Limousin breed were also found when comparing Holstein-Friesian breed with Limousin. Examples of consistently affected pathways are the P38 MAPK signaling and the Hypertrophy Model pathways (**Table 2**).

**Table 1:** The highest ranked pathways in skeletal muscle of Hereford compared to Limousin breed.

Pathway	Positive	Measured	Total	*Z* Score	P-value	Marbling
Hypertrophy Model	8	15	19	3.94	0.000	*
MAPK signaling pathway	34	124	167	3.51	0.002	*
Histone Modifications	13	35	43	3.41	0.000	-
IL-1 signaling pathway	15	41	54	3.17	0.003	*
P38 MAPK signaling pathway	10	27	36	2.98	0.006	*
Cardiac progenitor differentiation	11	33	54	2.72	0.006	-
T- Cell antigen Receptor signaling pathway	19	68	89	2.70	0.011	*
MicroRNAs in cardiomyocyte hypertrophy	20	73	102	2.67	0.006	*
Mitochondrial gene expression	5	11	23	2.66	0.006	*
Physiological and pathological hypertrophy of the heart	7	19	26	2.47	0.012	*
Insulin Signaling	27	111	157	2.41	0.012	*
Extracellular vesicle- mediated signaling in recipient cells	6	17	30	2.16	0.023	-
Toll-like receptor signaling pathway	18	71	92	2.15	0.032	*
Vitamin D metabolism	4	10	20	2.06	0.029	-
Alpha 6 Beta 4 signaling pathway	7	22	33	2.01	0.024	-

*Pathways previously known to be related to marbling.

The pathways are ranked based on Z score. Per pathway the following is listed; Positive = amount of genes differentially expressed, Measured = the amount of genes measured in the study, Total = the amount of genes in the pathway and P-value = the significance level.

**Table 2:** The highest ranked pathways in skeletal muscle of Holstein-Friesian compared to Limousin breed.

Pathway	Positive(*r*)	Measured(*n*)	Total	*Z* Score	P-value	Marbling
P38 MAPK signaling pathway	14	27	36	3.95	0.000	*
Quercetin and Nf-kB/AP-1 Induced Cell Apoptosis	7	10	26	3.77	0.000	-
Glycolysis and Gluconeogenesis	18	40	67	3.68	0.000	-
Hypertrophy Model	9	15	19	3.67	0.001	*
MAPK Signaling Pathway	42	124	167	3.48	0.000	*
Insulin Signaling	37	111	157	3.15	0.001	*
Eicosanoid Synthesis	8	15	38	3.04	0.000	-
Selenium Metabolism and Selenoproteins	11	26	48	2.63	0.009	-
IL1 and megakaryocytes in obesity	9	20	25	2.6	0.008	*
EGF/EGFR Signaling Pathway	34	110	156	2.5	0.019	-
Interferon type I signaling pathways	14	37	54	2.47	0.015	-
Cori Cycle	6	12	30	2.43	0.006	-
Integrated Cancer pathway	12	31	45	2.38	0.016	-
Myometrial Relaxation and Contraction Pathways	36	120	156	2.37	0.014	-
Pathogenic Escherichia coli infection	14	39	54	2.24	0.031	-

*Pathways previously known to be related to marbling.

The pathways are ranked based on Z score. Per pathway the following is listed; Positive = amount of genes differentially expressed, measured = the amount of genes measured in the study, Total = the amount of genes in the pathway and P-value = the significance level.

### Hypertrophy Model Pathway

**Figure 2:**
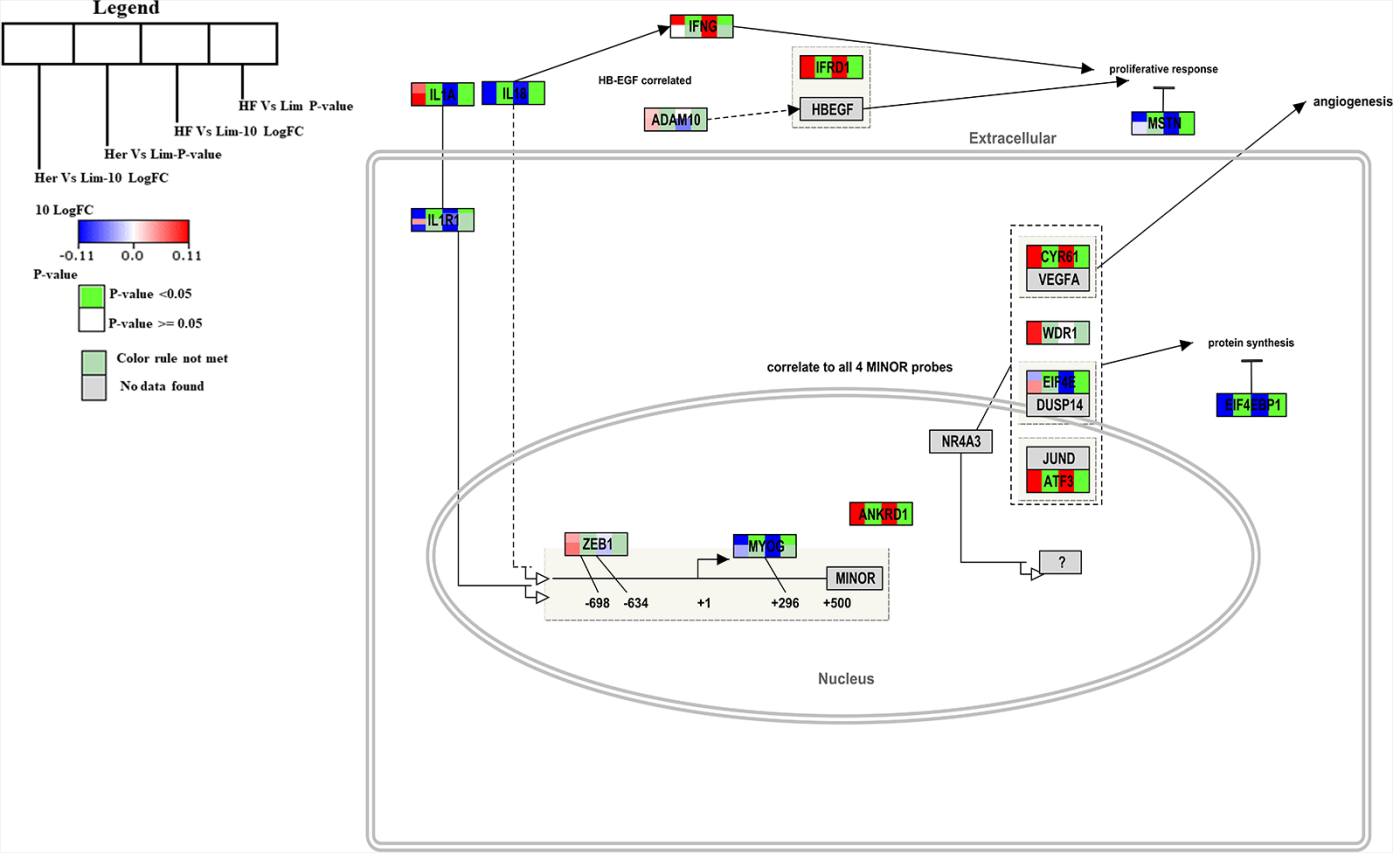
Skeletal muscle gene expression in Hereford and Holstein-Friesian vs Limousin visualized on the Hypertrophy model. In the hypertrophy model from WikiPathways (WP982) the changes in gene expression between Hereford and Holstein-Friesian with Limousin in skeletal muscle are visualized. The logFC (Hereford and Holstein-Friesian vs Limousin) is indicated with a color gradient (blue to red over white), i.e., blue represents a negative value (= lower expressed in Hereford and Holstein-Friesian) and red a positive value (= higher expressed in Hereford and Holstein-Friesian). The p-value is colored based on a rule, i.e. p-value < = 0.05 (= significant) is shown in green and p-value > 0.05 in white.

Muscle hypertrophy is known to increase the muscle mass, and is determined by increased protein mass per fiber which results from an increase of protein synthesis (Glass, 2005). In the *B. taurus* Hypertrophy model (http://www.wikipathways.org/instance/WP982) the overall gene expression was higher in skeletal muscle of Hereford and Holstein-Friesian compared to Limousin (**Figure 2**). The expression of the Il1a, Ifrd1, Cyr61, ATF3, and Ankrd1 genes were significantly higher in Hereford in this pathway and the Il18, Eif4ebp1, and Il1r1 genes were significantly lower in the model (p-value < 0.05). Among them was IL-1 which plays a significant role in lipid metabolism by regulating insulin levels under physiological conditions (Matsuki et al., 2003); Atf3 which works together with p38c in a common pathway in the intestine to regulate lipid metabolism and immune homeostasis (Chakrabarti et al., 2014); and Frd1, Cyr61, and Ankrd1 genes. Two of the seven genes were significantly lower expressed including Ef4ebp1 contributing to the development of obesity through increased adipogenesis and fat metabolism alterations (Le Bacquer et al., 2007) and Il18 gene. TNF and interleukin (IL)-1 may cause negative inotropic effects indirectly through activation or release of IL-18 (Mann, 2015).

### p38 MAPK Signaling Pathway

**Figure 3:**
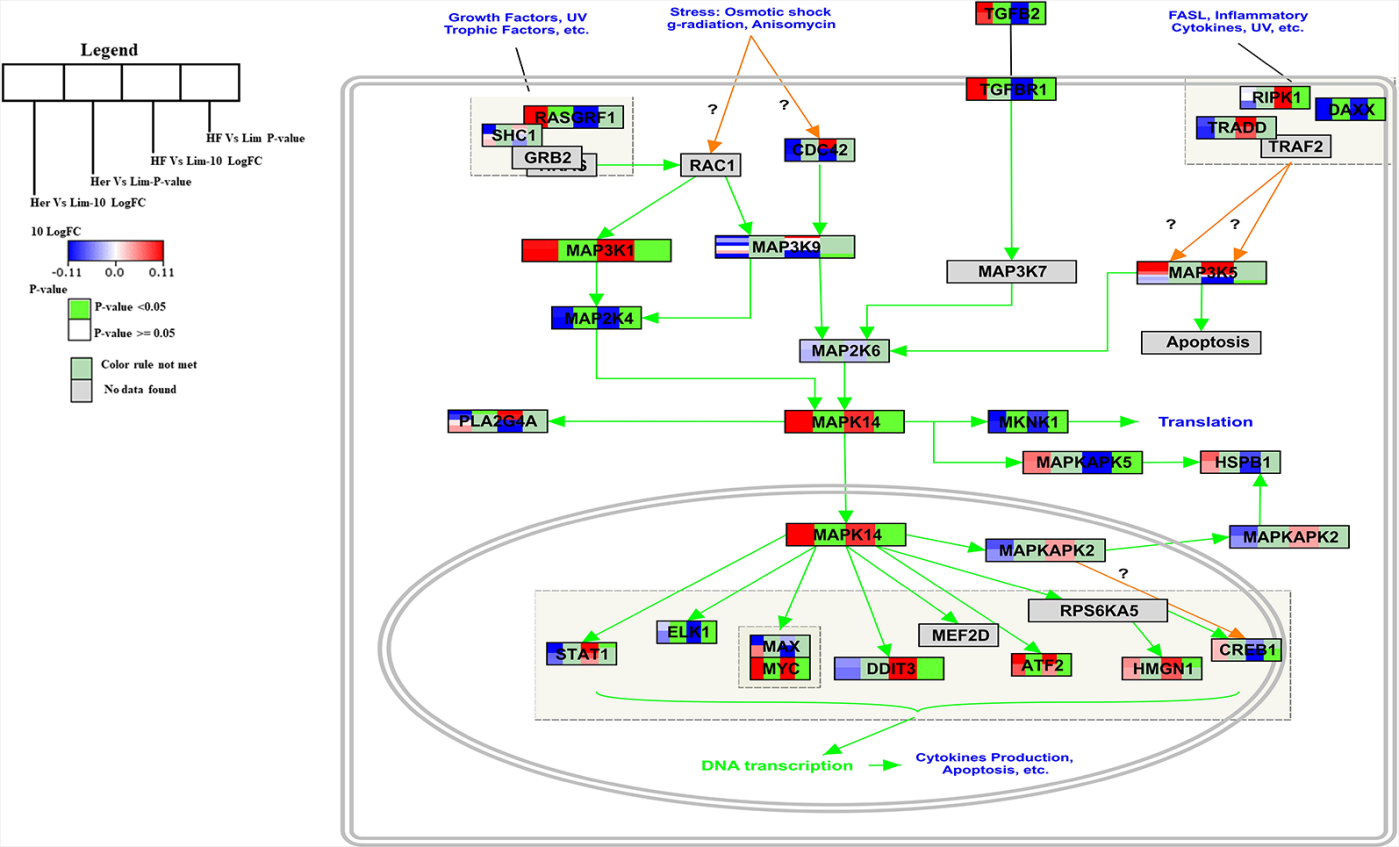
Skeletal muscle gene expression in Hereford and Holstein-Friesian vs Limousin visualized on the p38 MAPK signaling. In the p38 MAPK signaling pathway from WikiPathways (WP1037) the changes in gene expression between Hereford and Holstein-Friesian with Limousin in skeletal muscle are visualized. The logFC (Hereford and Holstein-Friesian vs Limousin) is indicated with a color gradient (blue to red over white), i.e., blue represents a negative value (= lower expressed in Hereford and Holstein-Friesian) and red a positive value (= higher expressed in Hereford and Holstein-Friesian). The p-value is colored based on a rule, i.e., p-value < = 0.05 (= significant) is shown in green and p-value > 0.05 in white.

The p38 Mitogen-activated protein kinase (p38 MAPK) signaling pathway has found to be responsible for transduction of extracellular signals to their intracellular targets in different types of cells, including skeletal muscle cells and which leads to several biological effects for example proliferation, differentiation, migration, growth, apoptosis, and more specifically to muscle cells, hypertrophy (Yu et al., 2010; Silva-Vignato et al., 2017). The p38 MAPK is one intracellular signaling pathway activated during the differentiation of myogenic cell lines and this pathway is a chief regulator of skeletal muscle development (Keren et al., 2006). The p38 MAPK signaling pathway is a well-known pathway that affects lipid metabolism (Zhang and Liu, 2002). In the *B. taurus* p38 MAPK signal pathway five of the seven genes present were significantly higher expressed in Hereford and Holstein-Friesian compared to Limousin (**Figure 3**).

### IL-1 Signaling Pathway

**Figure 4:**
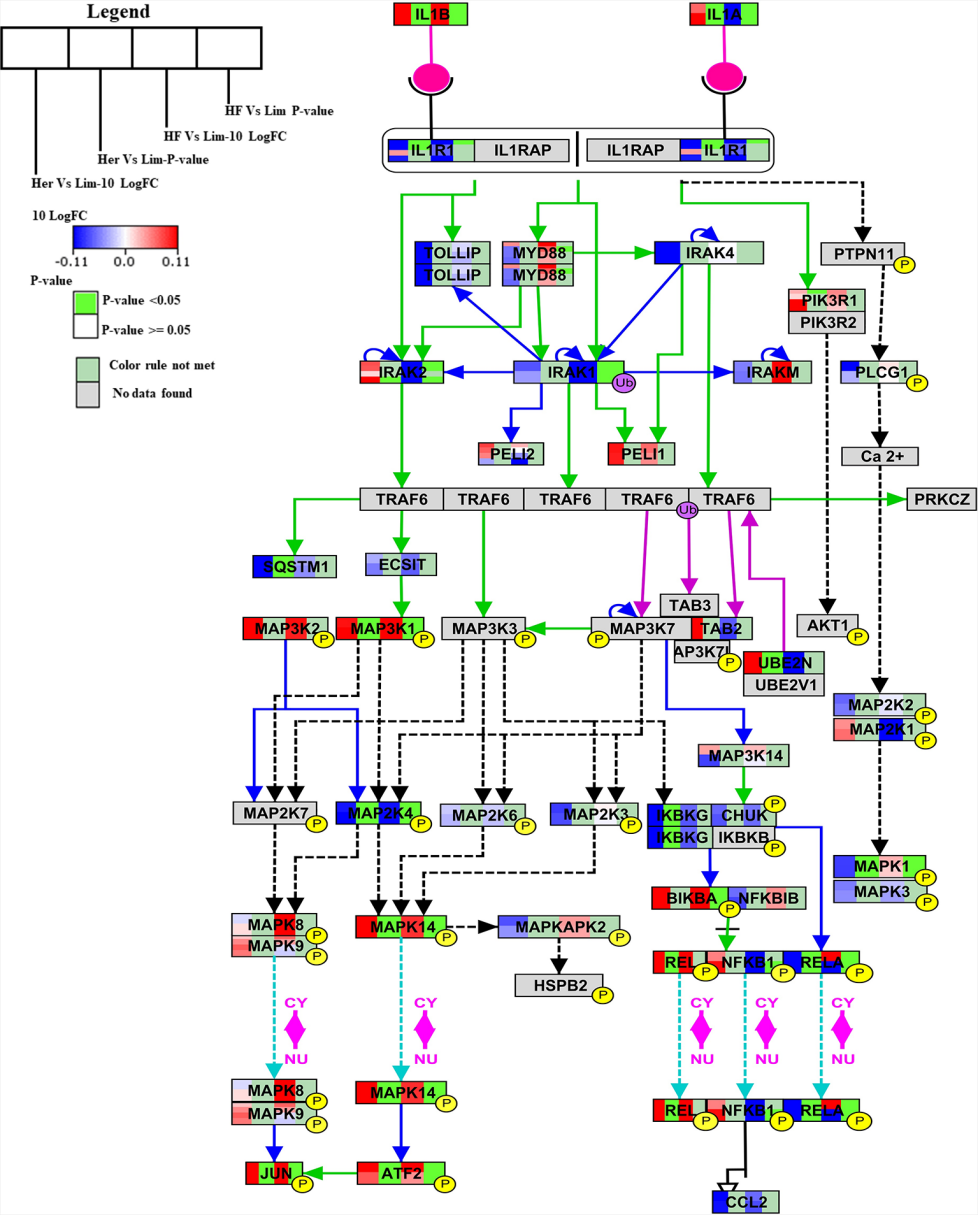
Skeletal muscle gene expression in Hereford and Holstein-Friesian vs Limousin visualized on IL-1 signaling. In the IL-1 signaling pathway from WikiPathways (WP3271) the changes in gene expression between Hereford and Holstein-Friesian with Limousin in skeletal muscle are visualized. The logFC (Hereford and Holstein-Friesian vs Limousin) is indicated with a color gradient (blue to red over white), i.e., blue represents a negative value (= lower expressed in Hereford and Holstein-Friesian) and red a positive value (= higher expressed in Hereford and Holstein-Friesian). The p-value is colored based on a rule, i.e., p-value < = 0.05 (= significant) is shown in green and p-value > 0.05 in white.

The IL-1 signal pathway is a major mediator of innate immune reactions.This pathway regulates extracellular and intracellular signaling of IL-1α or IL-1β including positive and negative-feedback mechanisms which strengthen or terminate the IL-1 response. In reply to ligand binding of the receptor, a complicated sequence of combinatorial phosphorylation and ubiquitination events lead to activation of nuclear factor kB signaling and the JNK and p38 mitogen-activated protein kinase pathways (Weber et al., 2010). The members of the *B. taurus* IL-1 signaling pathway (http://www.wikipathways.org/instance/WP3271), such as IL-1α, IL-1β, MAP3K1, UBE2N, MAPK14, REL, ATF2, and JUN were significantly up-regulated in Hereford breed versus Limousin breed (**Figure 4**). Among them IL-1α which was found to play a role as an inhibitor of the expression of peroxisome proliferator-activated receptor gamma (PPARG), a key transcriptional factor for adipocytes differentiation, (Um et al., 2011). IL-1β has been reported to inhibit adipocyte differentiation from preadipocytes and to reduce the lipid content in mature adipocytes (Simons et al., 2005). Some of aforementioned genes: MAP3K1, UBE2N, MAPK14, REL, ATF2 can directly bind to the peroxisome proliferator-activated receptor promoter and activate transcription to regulate adipocyte differentiation (Maekawa et al., 2010). The significantly down-regulated genes are: RELA, MAPK1, IKBKG, MAP2K4, SQSTM1, and IL1R1 (**Figure 4**). Some of them are known to participate in lipid metabolism processes; activation of p62/SQSTM1 and peroxisome proliferator-activated receptor gamma is induced by palmitate internalization, which triggers lipid metabolism and limits inflammation (Krausgruber et al., 2011).

### Insulin Signaling Pathway

**Figure 5:**
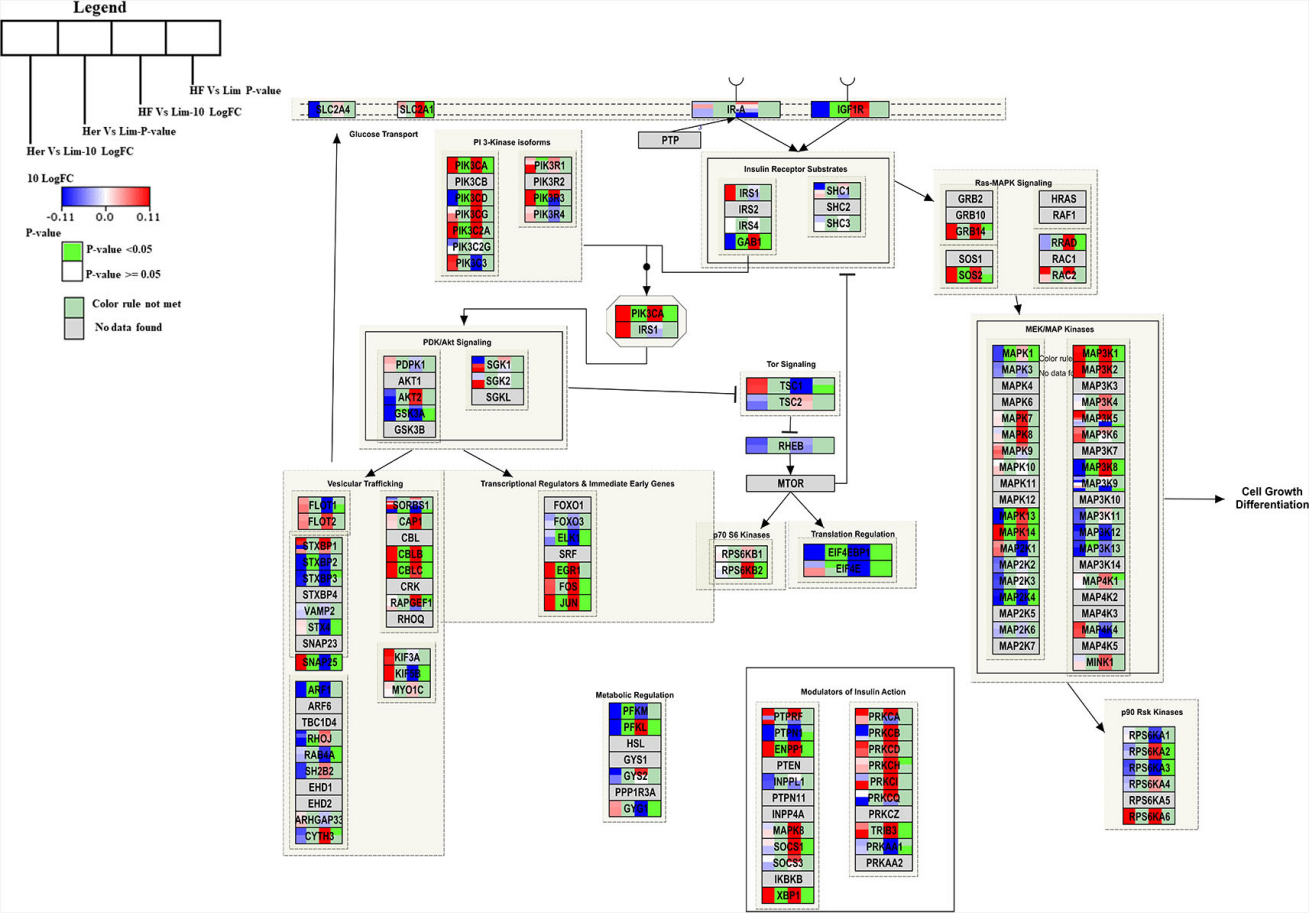
Skeletal muscle gene expression in Hereford and Holstein-Friesian vs Limousin visualized on the Insulin signal pathway. In the Insulin signaling pathway from WikiPathways (WP966) the changes in gene expression between Hereford and Holstein-Friesian with Limousin in skeletal muscle are visualized. The logFC (Hereford and Holstein-Friesian vs Limousin) is indicated with a color gradient (blue to red over white), i.e., blue represents a negative value (= lower expressed in Hereford and Holstein-Friesian) and red a positive value (= higher expressed in Hereford and Holstein-Friesian). The p-value is colored based on a rule, i.e. p-value < = 0.05 (= significant) is shown in green and p-value > 0.05 in white.

Genes engaged in the insulin signaling pathway regulate several aspects of cellular function, including most notably the regulation of cellular growth and maintaining glucose homeostasis (DeBosch and Muslin, 2008). For Hereford vs. Limousine comparison, twenty six genes present in the *B. taurus* Insulin signaling pathway (http://www.wikipathways.org/instance/WP966) showed significant expression differences. Fourteen genes were identified as up-regulated in Hereford breed (SOS2, PIK3R3, PIK3CA, PIK3C2A, CBLB, CBLC, SNAP25, JUN, EGR1, MAP3K1, MAPK14. ENPP1, and XBP1), and twelve were down-regulated in Hereford breed (PFKM, PFKL, ARF1, STXBP2, EIF4EBP1, PIK3CD, GAB1, IGF1R, MAPK1, MAPK13, MAP2K4, and ELK1) when compared to Limousin breed (**Figure 5**). The involvement of some of upregulated and downregulated genes in lipid accumulation processes were earlier confirmed, including;PIK3CA (Foukas et al., 2013), JUN (Guo et al., 2016), EGR1 (Singh et al., 2015), p38 MAPK lipid accumulation (Sun et al., 2012), NPP1 (Pan et al., 2011), XBP-1 (Zhao et al., 2012), 4E-BP1/2 (Singh et al., 2015), and IGF-1R (Freude et al., 2012).

### Gene Ontology Analysis

Pathway analysis gave an insight in the biological processes involved in marbling. However, only 69% of all measured genes are present in the investigated pathways from the *B. taurus* WikiPathways collection. In order to obtain a better insight in the biological role of the differentially expressed genes not present in WikiPathways a GO analysis was performed. (**Table 3**). This approach not only helps to unify the characteristics and functions of the genes but also to attain a broader perspective of the muscle physiological processes and their influence on meat quality related to marbling.

**Table 3:** The enriched processes found by GO-Analysis. A description of the process together with the positive gene number, Z Score, and P-value are given.

GOID	GO Name	GO Type	Gene Number	Z Score	P-value
**GO:0051092**	positive regulation of NF-kappa B transcription factor activity	Biological process	14	4.89	0.000
**GO:0009826**	unidimensional cell growth	Biological process	3	4.2	0.003
**GO:0090257**	regulation of muscle system process	Biological process	11	3.3	0.001
**GO:0051059**	NF-kappa B binding	Molecular function	5	3.3	0.002
**GO:0051781**	positive regulation of cell division	Biological process	8	3.28	0.001
**GO:0003009**	skeletal muscle contraction	Biological process	3	3.18	0.004
**GO:0045444**	fat cell differentiation	Biological process	13	3.01	0.000
**GO:0048009**	insulin-like growth factor receptor signaling pathway	Biological process	4	2.83	0.006
**GO:0050431**	transforming growth factor beta binding	Molecular function	3	2.82	0.008
**GO:0048741**	skeletal muscle fiber development	Biological process	3	2.26	0.020
**GO:0045598**	regulation of fat cell differentiation	Biological process	8	2.25	0.005
**GO:0007528**	neuromuscular junction development	Biological process	5	2.15	0.009

### Integrated Network of Altered Pathways With GO-Terms

**Figure 6:**
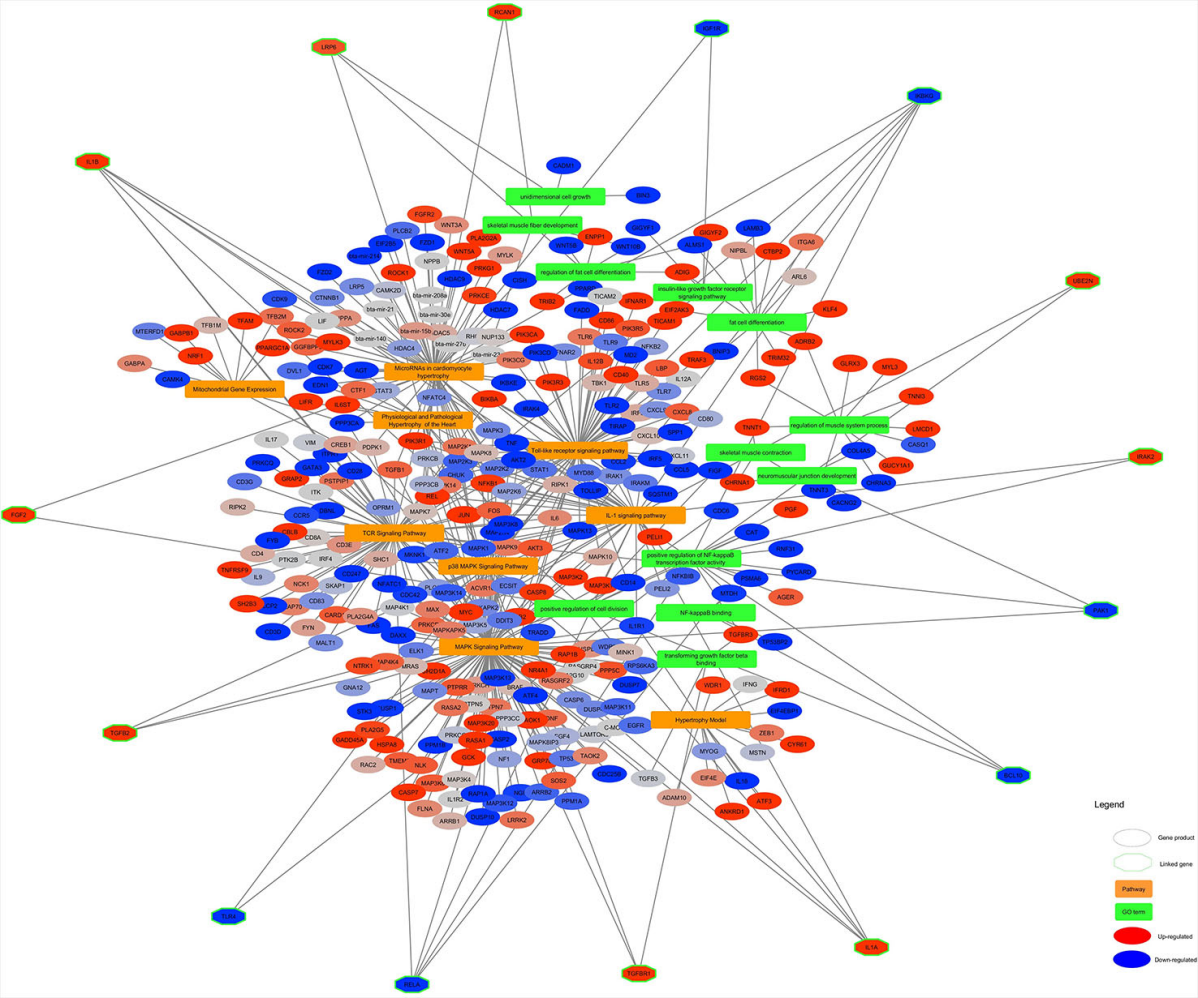
Altered pathways and GO terms in skeletal muscle of Hereford vs Limousin. In the network, pathways are shown in orange rectangles and GO Terms are shown in green rectangles. Differentially expressed genes are shown in red (= higher expressed in Hereford) and blue (= lower expressed in Hereford). Linked genes common between two process pathway and GO terms (green diamonds) are depicted with a hexagon shape.

The significant pathways and GO terms were merged together and shown in **Figure 6**. Some of the highly connected nodes are IL1α, TGFB2, PAK1, (as Pak1 deficiency led to upregulation of reverse cholesterol transporters in ApoE−/− mice in response to Western diet feeding, it might be suggested that Pak1 exerts a negative modulatory influence on these transporters and thereby might promote lipid retention in inflamed arteries which cause atherogenesis (Singh et al., 2015), TGFB2 (that TGF-β2 might control adipocyte differentiation in bone marrow stromal cells *in vivo* by inducing PPARγ phosphorylation. Whether Smad activation induced by TGFβ2 might play a role with MAPK in the inhibition of adipocyte differentiation induced by TGFβ2 *in vivo* requires more investigation (Ahdjoudj et al., 2005), IL1β, UBE2N, IRAK2, RCAN1, LRP6 (Treatment of LRP6 knockdown-Human mesenchymal stem cells with adipogenic supplements led to the accumulation of fat vacuoles, which was demonstrated by Oil Red O staining (Peröbner et al., 2012), IKBKG, TGFB3, RELA, BCL10, FGF2 (FGF-2 treatment of human preadipocytes also resulted in increased adipocyte differentiation, suggesting that this feature might be common to members of the fibroblast growth factor family, although FGF-1 was consistently the more potent adipogenic agent, particularly in cells from subcutaneous depots (Hutley et al., 2004), IGF1R, and TLR4 (TLR4 knockdown in H9C2 cardio myocytes decreases fatty acid-induced lipid accumulation (Dong et al., 2012) which are present in at least two different pathways and GO terms.

## Discussion

The aim of this study was to determine transcriptional profiles of high and low marbled beef with a focus on pathways of muscle cell origin that might play a role in the regulation of marbling development. The regulation of marbling is suggested to be the result of interaction of signaling pathways in muscle, fat, and intramuscular connective tissue (Hocquette, 2010). Identifying these processes with pathway analysis can help to decipher the key processes involved in marbling development. Pathway analysis revealed 17 pathways that were significantly different (z-score > 1.96) between well-marbled and lean marbled breeds. P38 MAPK signaling pathway well known to affects lipid metabolism and muscle development, was enriched when we compared gene expression in well and low marbling breeds. In addition, the signaling pathways “Hypertrophy Model”, “MicroRNAs in cardiomyocyte hypertrophy” and “Physiological and pathological hypertrophy of the heart” that play a role in tissue development were affected. Interestingly, the analyses also demonstrated that pathways related to immune response (IL signaling, TCR signaling, and Toll-like receptor signaling pathways) and insulin signaling, mitochondrial gene expression and vitamin D metabolism were enriched and might act together with pathways related to lipid metabolism. We explored regulatory pathways that control gene expression in bovine muscle and the relationships between gene expression and the marbling trait to identify markers that effect on marbling. A similar study done by (Hong et al., 2014) investigated the biological characteristics of differentially expressed genes in high marbled muscle in pig compared to a low marbled muscle. They indicated that the differentially expressed genes were clustered to three group related to energy metabolism, protein synthesis, and immune response in high marbling pigs. These finding suggested that the genes related to energy metabolism, protein synthesis, and immune response contribute to growth performance and meat quality. Our results also showed differentially expressed genes take part in these processes. The hypertrophy model pathway was found to be enriched with the highest Z score in the present study and during muscle hypertrophy there is an equilibrium between protein synthesis and degradation that might bring about protein deposition, and hence muscle growth. Together, these processes will lead to differences in muscle and fat deposition, and for this reason animals have different proportions of ribeye area and back fat thickness (Silva-Vignato et al.,2017). Mitogen-activated protein kinase (MAPK) signals have been shown to play a significant role in intracellular signaling associated with a variety of cellular activities including cell proliferation, differentiation, survival, and death (Yu et al., 2010). In mammalian cells, three MAPK families have been characterized: classical MAPK (also known as ERK), C-Jun N-terminal kinase/stress activated protein kinase and p38 kinase pathways (Zhang and Liu, 2002). Each mammalian MAPK signaling route comprises at least three components: a MAPK kinase kinase (MAP3K), a MAPK kinase (MAP2K), and a MAPK. Activated MAPKs phosphorylate various substrate proteins including transcription factors such as ATF2 and Jun (Kim & Choi, 2010). Philip and coworkers (Philip et al., 2005) discovered that the p38 MAPK played a key role in GDF-8-induced inhibition of proliferation and upregulation of the cyclin kinase inhibitor p21. In addition, their results showed a functional link between the p38 MAPK and GDF-8-activated Smad pathways, and identify an important role for the p38 MAPK in GDF-8’s function as a negative regulator of muscle growth (Philip et al., 2005). In comparative muscle transcriptome associated with carcass traits of Nellore cattle, Silva-Vignato and colleagues indicated that MAPK signaling pathway involved in muscle and fat deposition, which are economically important carcass traits for beef production (Silva-Vignato et al., 2017). The third pathway found in the present study was IL-1, the IL-1 family of cytokines includes 11 proteins encoded by 11 different genes and gene regulation of IL-1 signal is activation of MKK4, MKK3, and MKK6 gene that activate NF-κB and p38 MAPK pathways (Weber et al., 2010). These two signaling pathway are needed to upregulate the expression of the key E3 ligases, *MuRF1*, which mediate the inhibition of protein synthesis (Clarke et al., 2007). Moreover, the insulin-signal transduction pathway, which was another pathway identified in the present study, is a highly conserved pathway that regulates cellular growth and when insulin binding to its cell-surface receptor, insulin receptor, activates a complex intracellular signaling network through insulin substrate proteins and the canonical PI3K and ERK cascades (Hocquette et al., 2010). Interestingly, insulin signaling is one of important factors involved in muscle development since stimulation of glucose utilization in fat and muscle cells in calves is occurring by enhancing insulin intracellular signaling (Jovanović et al., 2017).

Currently, systems biology approaches have become one of the most effective manners to accelerate the genetic improvement of beef and dairy cattle herds (Kadarmideen, 2014). It allows the selection of desired characteristics through the use of transcriptome profiles. The development of high throughput data and bioinformatics tools allow the selection of superior breeds without wasting time and money, contributing to the widespread use of transcriptome analysis in beef cattle operations. Our study shows in cattle that integration of pathway expression profiles in a systems biology approach will contribute to a better understanding of the genes and regulatory processes involved in marbling. These novel insights can be used in the future to take into account when improving the meat quality in beef cattle. The molecular mechanisms which underlie fat content in muscle can provide vital information for the production of healthier beef for human consumption.

## Conclusions

The outcome of our research is the identification of biological pathways where we highlighted changed genes which are related with marbling in beef cattle. These results give a better understanding of mechanisms involving marbling in beef cattle, which is economically important carcasses trait for meat quality. Moreover, the genes involved in the *highlighted pathways can* potentially be utilized as an early biological marker for marbling fat content in breed-specific differences in growth performance and meat quality.

## Data Availability Statement

The datasets analyzed for this study can be found in the NCBI Gene Expression Omnibus (GEO) under the accession number GSE46411 and in the wikipathways.org knowledge base under the accession numbers: WikiPathways: WP2890, WikiPathways: WP2891, WikiPathways: WP2891, and WikiPathways: WP2901.

## Ethics Statement

Ethical review and approval was not required for the animal study because this study is just analysis.

## Author Contributions

ZR, SC, and CE conceptualized and designed the study. ZR, SC, MK, LE, and JM analyzed the data. ZR collected and assembled the data and wrote the manuscript and SC, CE, and TS contributed to reviewing and editing the manuscript and also read and approved the final manuscript.

## Conflict of Interest

The authors declare that the research was conducted in the absence of any commercial or financial relationships that could be construed as a potential conflict of interest.
